# Chronic Systemic Exposure to Low-Dose Rotenone Induced Central and Peripheral Neuropathology and Motor Deficits in Mice: Reproducible Animal Model of Parkinson’s Disease

**DOI:** 10.3390/ijms21093254

**Published:** 2020-05-04

**Authors:** Ikuko Miyazaki, Nami Isooka, Fuminori Imafuku, Jin Sun, Ryo Kikuoka, Chieko Furukawa, Masato Asanuma

**Affiliations:** Department of Medical Neurobiology, Okayama University Graduate School of Medicine, Dentistry and Pharmaceutical Sciences, Okayama 700-8558, Japan; pi3s34bh@s.okayama-u.ac.jp (N.I.); p9lz3yiz@s.okayama-u.ac.jp (F.I.); pwdz7nma@s.okayama-u.ac.jp (J.S.); p32m0bvx@s.okayama-u.ac.jp (R.K.); pknp1481@s.okayama-u.ac.jp (C.F.); asachan@cc.okayama-u.ac.jp (M.A.)

**Keywords:** rotenone, Parkinson’s disease, dopaminergic neuron, dorsal motor nucleus of the vagus, myenteric plexus, neurodegeneration, α-synuclein, motor deficit

## Abstract

Epidemiological studies demonstrated that pesticide exposure, such as rotenone and paraquat, increases the risk of Parkinson’s disease (PD). Chronic systemic exposure to rotenone, a mitochondrial complex I inhibitor, could reproduce many features of PD. However, the adoption of the models is limiting because of variability in animal sensitivity and the inability of other investigators to consistently reproduce the PD neuropathology. In addition, most of rotenone models were produced in rats. Here, we tried to establish a high-reproducible rotenone model using C57BL/6J mice. The rotenone mouse model was produced by chronic systemic exposure to a low dose of rotenone (2.5 mg/kg/day) for 4 weeks by subcutaneous implantation of rotenone-filled osmotic mini pump. The rotenone-treated mice exhibited motor deficits assessed by open field, rotarod and cylinder test and gastrointestinal dysfunction. Rotenone treatment decreased the number of dopaminergic neuronal cells in the substantia nigra pars compacta (SNpc) and lesioned nerve terminal in the striatum. In addition, we observed significant reduction of cholinergic neurons in the dorsal motor nucleus of the vagus (DMV) and the intestinal myenteric plexus. Moreover, α-synuclein was accumulated in neuronal soma in the SNpc, DMV and intestinal myenteric plexus in rotenone-treated mice. These data suggest that the low-dose rotenone mouse model could reproduce behavioral and central and peripheral neurodegenerative features of PD and be a useful model for investigation of PD pathogenesis.

## 1. Introduction

Parkinson’s disease (PD) is a common neurodegenerative disease. Loss of nigrostriatal dopaminergic neurons causes motor symptoms, such as akinesia/bradykinesia, tremor, rigidity, and postural instability. Braak et al. [1] reported that PD pathology, Lewy bodies and Lewy neuritis, within the central nervous system (CNS), appeared first in the brainstem (dorsal motor nucleus of the vagus (DMV)), and then spread upward progressively through the substantia nigra, eventually leading to motor dysfunction, to reach the cerebral cortex. In addition, several reports have demonstrated that PD pathology is also detected within the enteric nervous system (ENS) [2,3,4]. Constipation is a well-known non-motor symptom in PD, which precedes motor symptoms by 10–20 years [5,6,7]. Therefore, it has been hypothesized that PD pathology propagates from the ENS to the CNS via vagal nerve [8,9,10] although the pathogenesis in sporadic PD remains unknown.

To clarify the mechanism of neurodegeneration in PD and to develop treatment to slow or stop PD progression, there is a great need for experimental models which exhibit neurological features of PD. For the past several decades, researchers reported various animal models using environmental or synthetic neurotoxins or expressing familial PD-related gene mutations [11,12]. Especially, models exposed to rotenone, a commonly used pesticide [13,14], have received most attention since Greenamyre and his colleagues reported that chronic exposure to rotenone could reproduce the anatomical, neurochemical, behavioral, and neuropathological features of PD [15,16]. In addition, importance of rotenone models is strengthened by epidemiological studies suggesting that pesticide exposure, particularly rotenone and paraquat, increases the risk of PD [17]. Moreover, several studies demonstrated that rotenone induced neuropathological change not only in the CNS but also ENS in animals [18,19,20]. Despite these advantage of rotenone models, the adoption of the models, in particular rotenone infusion models, is limiting because of variability in animal sensitivity and the inability of other investigators to consistently reproduce the PD neuropathology. To dissolve this issue, other routes of administration of rotenone have been developed: Daily intraperitoneal (i.p.) injection for up to 60 days [21], daily oral administration for 56 days [22], daily intranasal inoculation for 30 days [23] and daily intragastric administration for 5 days a week for 1.5 and 3 months [19]. However, these administrations still have disadvantages: High mortality of animals, difficulty in method, and health hazard of investigators exposed to the toxin by daily administration. In addition, most of rotenone models were produced in rats [24]. As mentioned above, the cause of sporadic PD remains unknown, but both genetic and environmental factors are thought to contribute to PD pathogenesis. Therefore, rotenone treatment in genetic mouse models of PD could be useful animal models to investigate possible interaction between pesticide exposure and genetic defects. We tried to develop a novel rotenone-treated mouse model by chronic systemic infusion of rotenone using osmotic mini pump. Recently, we reported chronic exposure to a low dose of rotenone (2.5 mg/kg/day) for 4 weeks exhibited neurodegeneration in the substantia nigra pars compacta (SNpc) and intestinal myenteric plexus [25].

Here, we re-evaluate the low-dose rotenone-treated mouse model by behavioral assessment and neuropathological analysis to establish a high-reproducible parkinsonian model in C57BL/6J mice, which is a most common strain for genetically modified animal. The rotenone-treated mice exhibited motor deficits, gastrointestinal (GI) dysfunction, and neurodegeneration accompanied with intracellular α-synuclein (α-Syn) accumulation in the nigrostriatal dopaminergic neurons, cholinergic neurons in the DMV and intestinal myenteric plexus.

## 2. Results

### 2.1. Chronic Exposure to Low-Dose Rotenone Induced Motor Deficits in Mice

**Figure 1:**
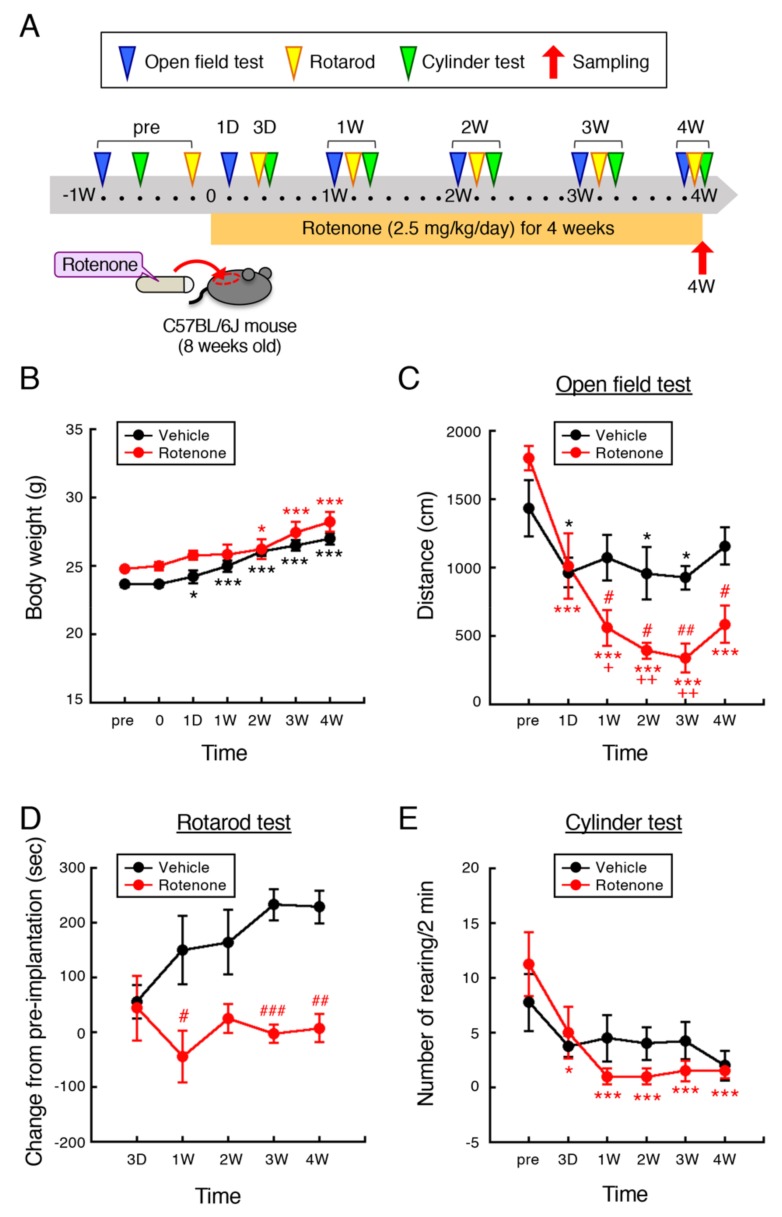
Chronic exposure to low-dose rotenone induced motor deficits in mice. (**A**) Schematic illustration of the experimental protocol. Male C57BL/6J mice (8 weeks old) were injected subcutaneously with rotenone (2.5 mg/kg/day) for 4 weeks using an osmotic mini pump. Behavioral assessment including open field, rotarod and cylinder test were performed at 1–6 days before (pre), 1, 3 days, 1, 2, 3, and 4 weeks after implantation of rotenone-filled pump. For immunohistochemistry and mitochondrial complex I activity analysis, tissue samples were collected at 4 weeks after implantation. (**B**) Change in body weight of mice. (**C**) Locomotor activity was assessed as total distance (cm) for 5 min using open field apparatus (50 × 50 × 35 cm). (**D**) Motor function was assessed by fixed-speed rotarod test (24 rpm). Change in time latency (s) from pre-implantation of pump was indicated. (**E**) Number of rearing with forelimbs contacts for 2 min was measured using transparent plastic cylinder (diameter: 13 cm, height: 16 cm). Each value is the mean ± SEM (*n* = 4). * *p* < 0.05, ** *p* < 0.01, *** *p* < 0.001 vs. value at pre-implantation, ^+^ *p* < 0.05, ^++^ *p* < 0.01 vs. value at 1 day after implantation, # *p* < 0.05, ## *p* < 0.01, ### *p* < 0.001 vs. the vehicle-treated control group (repeated measures ANOVA followed by Fisher’s LSD test).

The experimental protocol is outlined in Figure 1A. Male C57BL/6J mice (8 weeks old) were injected subcutaneously with rotenone (2.5 mg/kg/day) for 4 weeks using an osmotic mini pump. Behavioral assessment including open field, rotarod and cylinder test were performed at 1–6 days before (pre), 1, and 3 days, and 1, 2, 3, and 4 weeks after implantation of vehicle- or rotenone-filled pump. After 4-week rotenone treatment, we collected tissue samples for immunohistochemistry and mitochondrial complex I activity analysis.

#### 2.1.1. Body Weight

Body weight of control and rotenone-treated mice increases in proportion to their growth. The increase in body weight of rotenone-treated mice was not observed 1 day and 1 week after implantation of rotenone-filled pump (Figure 1B). However, rotenone treatment did not induce weight loss at any time point. In addition, there was no difference in the survival rate between control and rotenone-treated mice during experimental period (data not shown).

#### 2.1.2. Open Field Test

To examine the effect of rotenone exposure on locomotor activity of mice, total distance moved was measured by open field test. The total distance of control and rotenone-treated mice was significantly decreased 1 day after implantation of pump compared with the pre-implantation. Aggravation of decrease in locomotor activity was observed in rotenone-treated mice, but not control, in the time dependent manner (Figure 1C).

#### 2.1.3. Rotarod Test

To evaluate motor coordination and balance, rotarod test was performed with fixed speed (24 rpm, cutoff time 300 s). Because of difference in walking ability of mice on the rotating rod, data was shown as change from value at pre-implantation of pump (Figure 1D). At 3 day after pump implantation, there was no difference between control and rotenone-treated groups. In vehicle-treated control mice, time of walking on the rotating rod was extended, but not in rotenone-treated mice.

#### 2.1.4. Cylinder Test

To evaluate motility of both forelimbs and hindlimbs, cylinder test was performed [26]. Because rotenone affects motor behavior bilaterally, the number of rearing with forelimbs contacts against the wall of plastic cylinder was measured for 2-min period irrespective of right and left paw. There was no change in the number of rearing in control mice at any time point. On the other hand, the number of rearing with forelimb contacts was significantly decreased in rotenone-treated mice (Figure 1E).

### 2.2. Chronic Exposure to Low-Dose Rotenone Induced Nigrostriatal Dopaminergic Neurodegeneration in Mice

**Figure 2:**
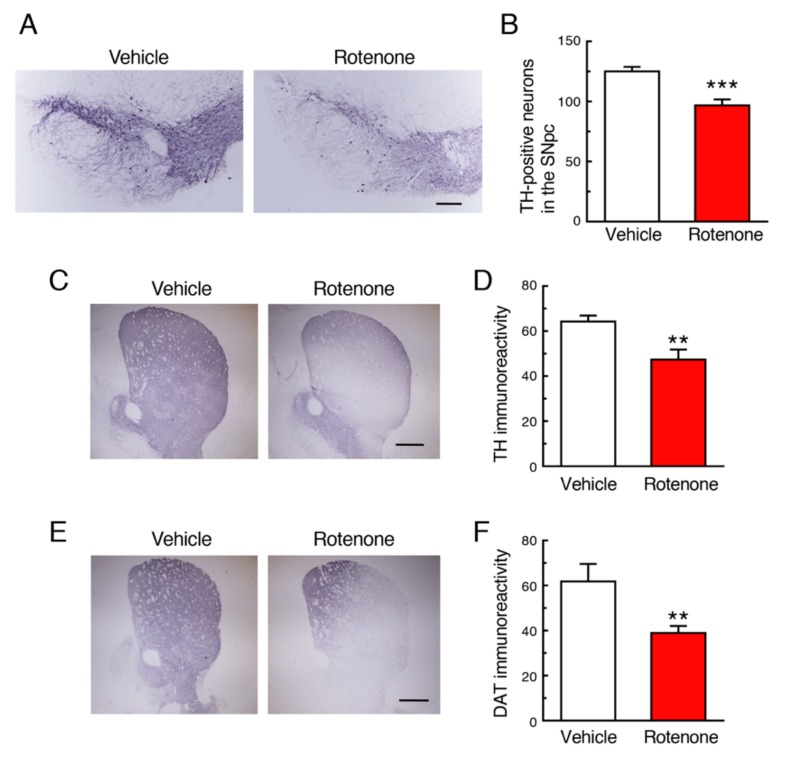
Chronic exposure to low-dose rotenone induced nigrostriatal dopaminergic neurodegeneration in mice. (**A**,**C**) Representative photomicrographs of immunohistochemistry for tyrosine hydroxylase (TH) in the substantia nigra pars compacta (SNpc) (**A**) and striatum (**C**) of mice 4 weeks after rotenone treatment. Scale bar = 200 µm (**A**), 500 µm (**C**). (**B**) Changes in the number of TH-positive nigral neurons. (**D**) Changes in the signal intensity of TH immunoreactivity in the striatum. (**E**) Representative photomicrographs of immunohistochemistry for dopamine transporter (DAT) in the striatum of mice 4 weeks after rotenone treatment. Scale bar = 500 µm. (**F**) Changes in the signal intensity of DAT immunoreactivity in the striatum. Each value is the mean ± SEM (*n* = 4). ** *p* < 0.01, *** *p* < 0.001 vs. the vehicle-treated control group (two-tailed unpaired independent t-test).

Chronic subcutaneous treatment with a low dose of rotenone (2.5 mg/kg/day) for 4 weeks significantly decreased the number of tyrosine hydroxylase (TH)-positive dopaminergic neurons in the SNpc (Figure 2A,B) and signal intensity of TH in the striatum (Figure 2C,D). In addition, nerve terminal degeneration of dopaminergic neurons in rotenone-treated mice was examined by immunostaining for dopamine transporter (DAT) using striatal sections. The striatal DAT-positive signal was also significantly decreased by rotenone treatment (Figure 1E,F).

### 2.3. Chronic Exposure to Low-Dose Rotenone Induced Cholinergic Neurodegeneration in the DMV in Mice

**Figure 3:**
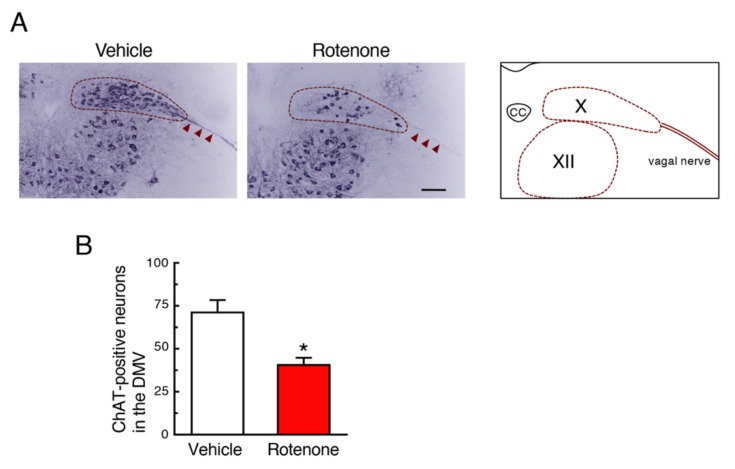
Chronic exposure to low-dose rotenone induced cholinergic neurodegeneration in the dorsal motor nucleus of the vagus (DMV) of mice. (**A**) Representative photomicrographs of immunohistochemistry for choline acetyltransferase (ChAT) in the DMV of mice 4 weeks after rotenone treatment. Scale bar = 100 µm. CC: central canal, X: DMV, XII: hypoglossal nucleus. Arrowheads: Vagal nerve. (**B**) Changes in the number of ChAT-positive neurons in the DMV. Each value is the mean ± SEM (*n* = 4). * *p* < 0.05 vs. the vehicle-treated control group (two-tailed unpaired independent *t*-test).

Next, we examined effects of rotenone exposure on cholinergic neurons in the DMV, where PD-related pathology was observed. Treatment with a low dose of rotenone significantly decreased the choline acetyltransferase (ChAT)-positive signals in the DMV and vagal nerve (Figure 3A,B). The ChAT-positive signals in the hypoglossal nucleus of rotenone-treated mice were almost same as control group (Figure 3A).

### 2.4. Chronic Exposure to Low-Dose Rotenone Induced Neurodegeneration in the Intestinal Myenteric Plexus in Mice

**Figure 4:**
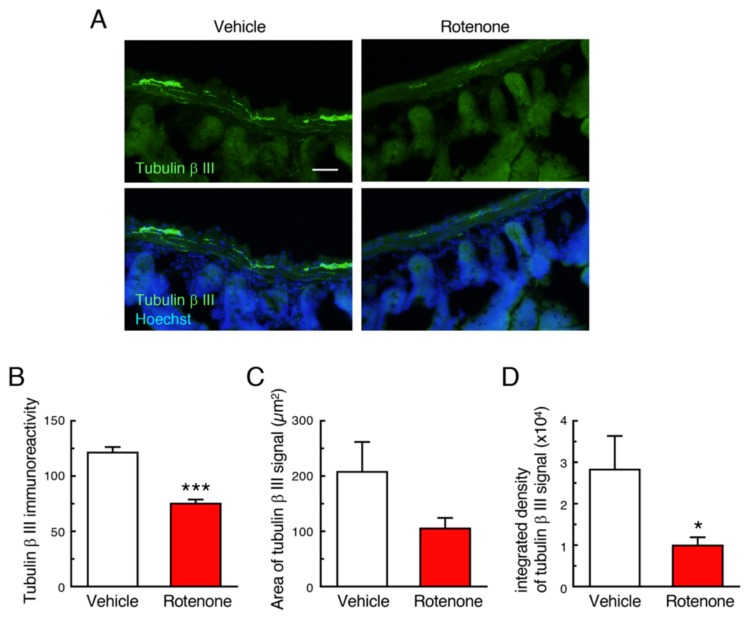
Chronic exposure to low-dose rotenone induced neurodegeneration in the intestinal myenteric plexus in mice. (**A**) Representative photomicrographs of immunohistochemistry for Tubulin β III in the intestine of mice. Green: Tubulin β III-positive neurons. Blue: nuclear staining with Hoechst 33342. Scale bar = 50 µm. (**B**–**D**) Quantitation of Tubulin β III-positive signals in the intestine. (**B**) Signal intensity of Tubulin β III immunoreactivity in the myenteric plexus. (**C**) Area of Tubulin β III-positive myenteric plexus, (**D**) Integrated density of Tubulin β III immunoreactivity. Each value is the mean ± SEM (*n* = 4). * *p* < 0.05, *** *p* < 0.001 vs. the vehicle-treated control group (two-tailed unpaired independent t-test).

To examine effects of rotenone exposure on the intestinal myenteric plexus in mice, we performed immunostaining of the neuronal marker, Tubulin β III. In vehicle-treated group, apparent Tubulin β III-positive signals were detected in the intestinal myenteric plexus (Figure 4A). In contrast, rotenone treatment significantly decreased the Tubulin β III immunoreactivity in the intestinal myenteric plexus (Figure 4A–D).

### 2.5. Chronic Exposure to Low-Dose Rotenone Induced GI Dysfunction in Mice

**Figure 5:**
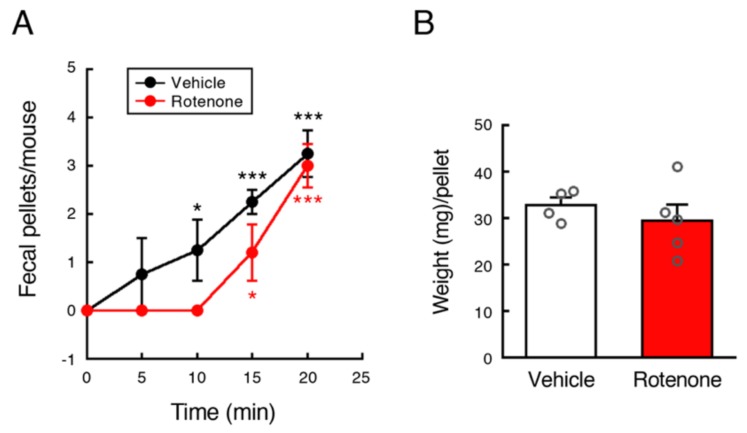
Chronic exposure to low-dose rotenone induced GI dysfunction in mice. Fecal output was measured at 4 weeks after implantation of vehicle- or rotenone-filled pump. (**A**) Time course of fecal outputs over 20 min. Each value is the mean ± SEM (Vehicle: *n* = 4; Rotenone: *n* = 5). (**B**) Weight (mg) of feces per pellet. Values are presented as dot blots with individual value plus bar with mean ± SEM (Vehicle: *n* = 4; Rotenone: *n* = 5). * *p* < 0.05, *** *p* < 0.001 vs. value at 0 min (one-way ANOVA followed by Fisher’s LSD test).

To examine effects of rotenone exposure on GI function in mice, fecal output was measured at 4 weeks after implantation of vehicle- or rotenone-filled pump. There was no change in total pellets produced during 20 min between vehicle- and rotenone-exposed mice. Vehicle-injected control mice defecated constantly every 5 min. On the other hand, all rotenone-treated mice took 15 min until first fecal output (Figure 5A). Further, weight of fecal pellets produced by rotenone-treated mice was varied in contrast to control mice (Figure 5B).

### 2.6. Chronic Exposure to Low-Dose Rotenone Induced α-Syn Accumulation in Dopaminergic Neurons in the SNpc of Mice

**Figure 6:**
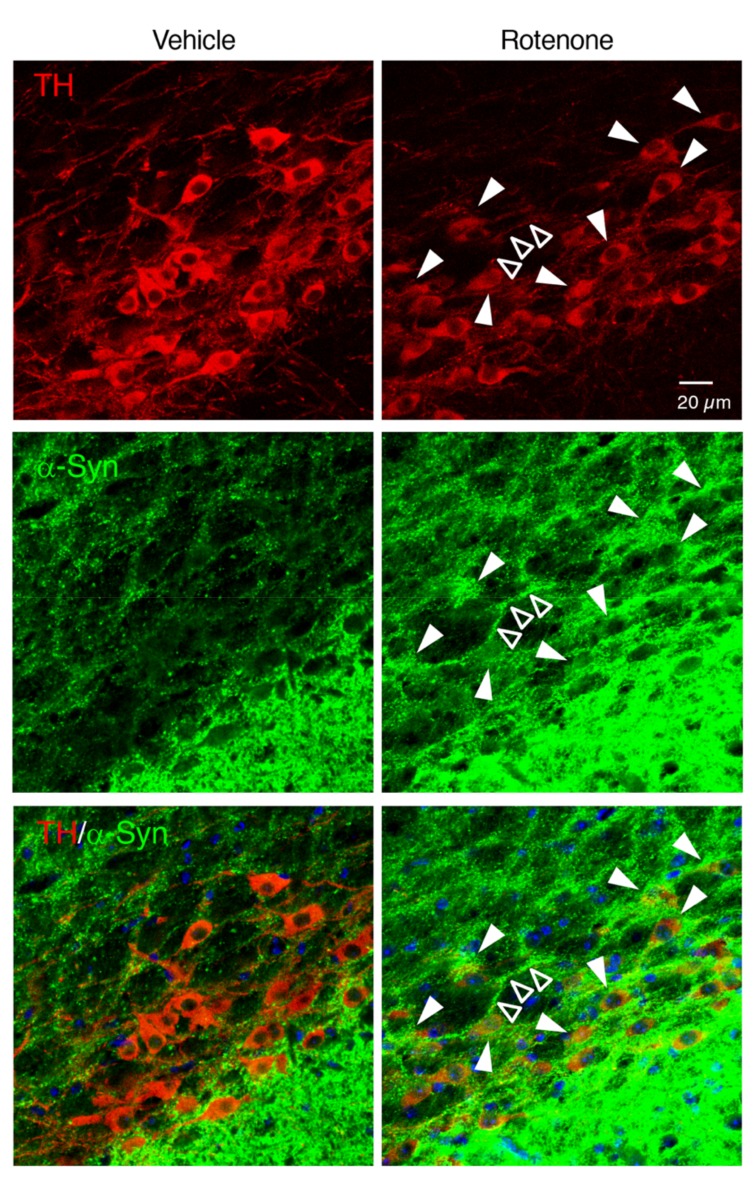
Chronic exposure to low-dose rotenone induced α-synuclein (α-Syn) accumulation in dopaminergic neurons in the SNpc of mice. Representative photomicrographs of TH and α-Syn double immunostaining in the SNpc of mice. Red: TH-positive neurons. Green: α-Syn. Blue: nuclear staining with Hoechst 33342. Solid arrowheads: increased α-Syn signals in TH-positive neuronal soma. Open arrowheads: α-Syn-positive neurite. Scale bar = 20 µm.

To examine whether chronic rotenone exposure induced α-Syn accumulation in the nigral dopaminergic neurons, we performed double immunostaining of TH and α-Syn. Rotenone treatment increased the α-Syn signals in the soma and neurites of TH-positive neurons in the SNpc (Figure 6).

### 2.7. Chronic Exposure to Low-Dose Rotenone Induced α-Syn Accumulation in the Cholinergic Neurons in the DMV of Mice

**Figure 7:**
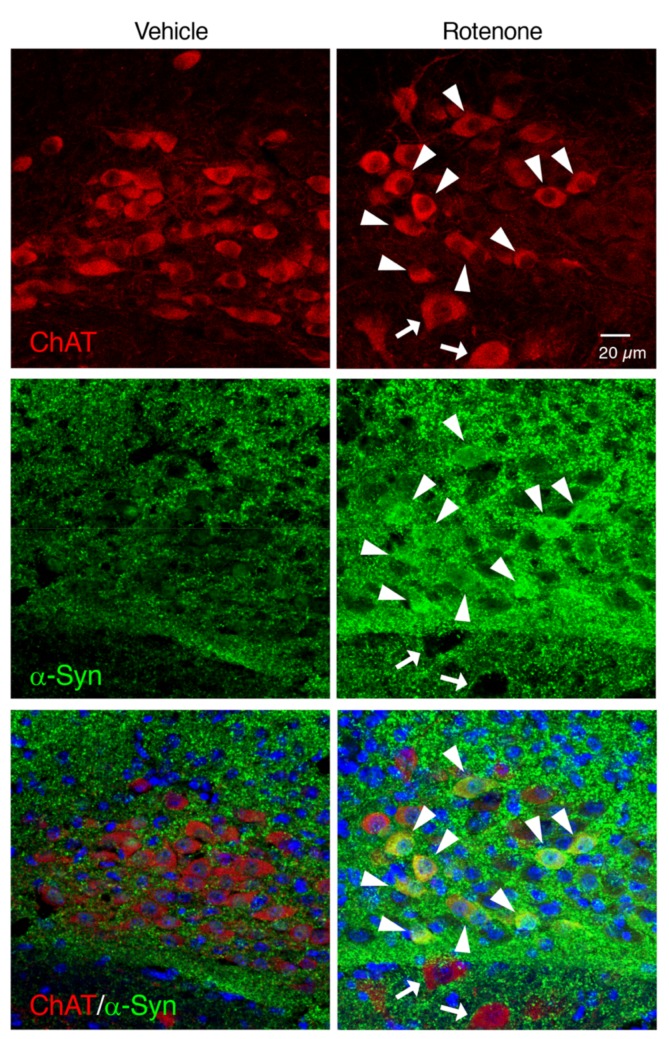
Chronic exposure to low-dose rotenone induced α-Syn accumulation in the cholinergic neurons in the DMV of mice. Representative photomicrographs of ChAT and α-Syn double immunostaining. Red: ChAT-positive neurons. Green: α-Syn. Blue: nuclear staining with Hoechst 33342. Solid arrowheads: increased α-Syn signals in neuronal soma in the DMV. Arrows: α-Syn-negative neurons in the hypoglossal nucleus. Scale bar = 20 µm.

To examine whether chronic rotenone exposure induced α-Syn accumulation in the cholinergic neurons in the DMV, we performed double immunostaining of ChAT and α-Syn. Apparent α-Syn-positive signals were detected in the ChAT-positive neurons in the DMV of rotenone-treated mice (Figure 7).

### 2.8. Rotenone-Induced α-Syn Accumulation Was Not Observed in the Cholinergic Neurons in the Hypoglossal Nucleus

**Figure 8:**
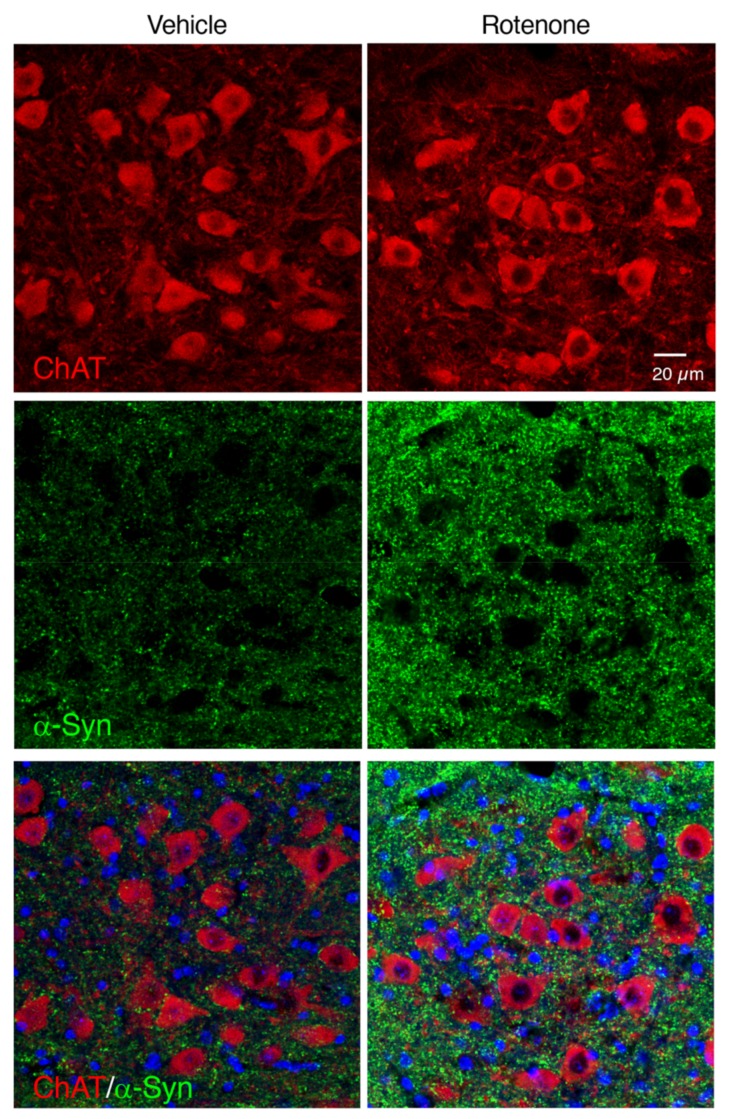
Changes in α-Syn-positive signals in the hypoglossal nucleus of rotenone-treated mice. Representative photomicrographs of ChAT and α-Syn double immunostaining. Red: ChAT-positive neurons. Green: α-Syn. Blue: nuclear staining with Hoechst 33342. Scale bar = 20 µm.

In contrast to the DMV, intracellular α-Syn accumulation was not observed in the cholinergic neurons in the hypoglossal nucleus of rotenone-treated mice (Figure 8).

### 2.9. Chronic Exposure to Low-Dose Rotenone Induced α-Syn Accumulation in the Intestinal Myenteric Plexus of Mice

**Figure 9:**
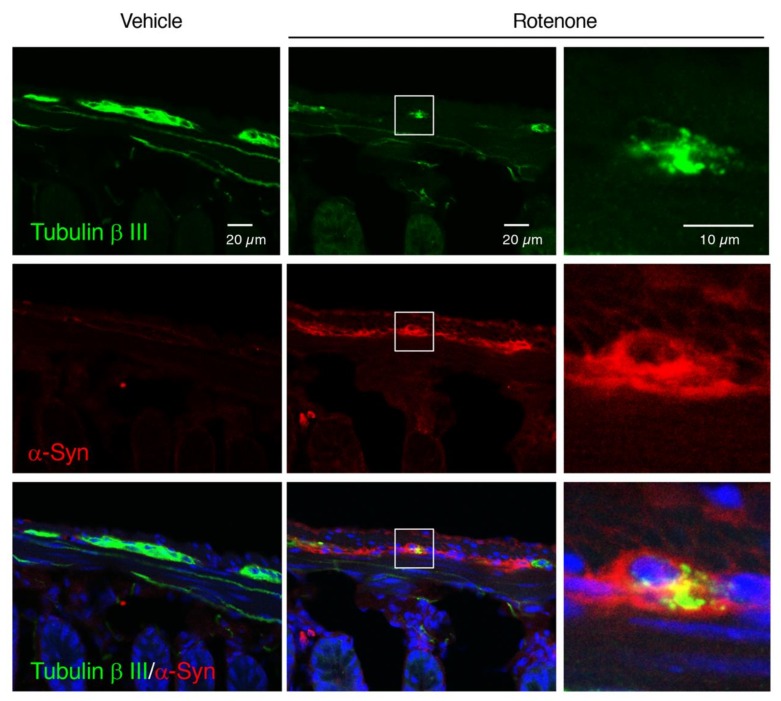
Chronic exposure to low-dose rotenone induced α-Syn accumulation in the intestinal myenteric plexus of mice. Representative photomicrographs of Tubulin β III and α-Syn double immunostaining. Green: Tubulin β III. Red: α-Syn. Blue: nuclear staining with Hoechst 33342. (Left, Center) Scale bar = 20 µm. (Right) Magnified photographs of white boxes. Scale bar = 10 µm.

To examine whether chronic rotenone exposure induced α-Syn accumulation in the myenteric plexus in the small intestine of mice, we performed double immunostaining of Tubulin β III and α-Syn. Rotenone exposure dramatically increased α-Syn-positive signals in the myenteric plexus. We also confirmed α-Syn accumulation in soma of Tubulin β III-positive neurons in rotenone-treated mice (Figure 9).

### 2.10. Mitochondrial Complex I Activity Was Not Decreased by Chronic Exposure to Low-Dose Rotenone

**Figure 10:**
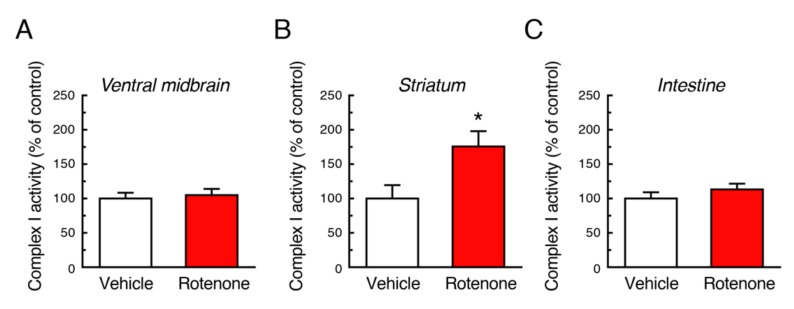
Changes in mitochondrial complex I activity after chronic exposure to low-dose rotenone in mice. Complex I activity was measured in the ventral midbrain (**A**), striatum (**B**) and intestine (**C**) 4 weeks after rotenone treatment. Each value is the mean ± SEM (Vehicle: *n* = 4; Rotenone: *n* = 5). * *p* < 0.05 vs. the vehicle-treated control group (two-tailed unpaired independent t-test).

Rotenone is a mitochondrial complex I inhibitor. To examine effects of long-term exposure to rotenone on mitochondrial function, we measured complex I activity using tissue samples of ventral midbrain, striatum and intestine. Chronic rotenone exposure did not decrease mitochondrial complex I activity in both CNS and ENS. In the striatum, rotenone treatment rather increased complex I activity (Figure 10).

## 3. Discussion

Previous several studies reported rotenone treatment in C57BL mice produced PD pathology [19,22,27]. Inden et al. [22] demonstrated daily oral administration of rotenone at 30 mg/kg for 28 days or 56 days caused specific nigrostriatal dopaminergic neurodegeneration and behavioral impairment. In addition, Richter et al. [27] reported that daily subcutaneous injection of rotenone (5 mg/kg) decreased spontaneous motor activity but failed to produce neurodegeneration of dopamine neurons. A noteworthy experiment was performed by Pan-Montojo et al. [19]. They reported that daily intragastric administration of rotenone (5 mg/kg) by using a stomach tube reproduced PD pathology in mice [19]. The model mice exhibited α-Syn accumulation in the ENS, the DMV, the intermediolateral nucleus of the spinal cord and the SN. These studies seem to provide an evidence that C57BL mice are also available for rotenone models as well as rats used in many previous experiments [24]. However, these administrations still have concern: High mortality of animals, difficulty in method, or health hazard of investigators exposed to the toxin by daily administration. Therefore, we performed chronic rotenone infusion by subcutaneous implantation of rotenone-filled pump because continuous exposure to rotenone using mini pump would be important and extremely simple. In our previous study, it is reported that subcutaneous administration of rotenone (50 mg/kg/day) for 3 or 6 months induced central and peripheral neurodegeneration in C57BL mice [28]. However, the dose of rotenone (50 mg/kg/day) was extremely high; rotenone treatment induced high mortality. Recently, we reported a new rotenone model using a lower dose of rotenone (2.5 mg/kg/day) compared with previous reports [19,22,27,28], corresponding to the environmental exposure levels of rotenone via pesticides. The rotenone treatment paradigm was designed based on the first remarkable report that chronic systemic rotenone exposure using osmotic mini pumps recapitulated many features of PD in Lewis rats [15]. The low-dose rotenone-treated mice exhibited neurodegeneration in the SNpc and intestinal myenteric plexus [25]. In addition, upregulation of antioxidative property by oral administration of coffee components inhibited rotenone-induced neurodegeneration not only in the CNS but also ENS [25].

In this study, we re-evaluated whether the low-dose rotenone model mice could reproduce features of PD. First, we evaluated effects of rotenone treatment on body weight and motor activity. In the previous studies, rotenone treatment induced obvious weight loss [16,29,30] and high mortality [22,27,28]. On the other hand, our rotenone-treated mice did not exhibit weight loss at any time point; the survival rate of rotenone-treated mice was not different from control group, and it was not changed during 4-week experimental period. The zero mortality is different from rat model and extremely important because that leads to stability of rotenone sensitivity. Furthermore, simple administration of pump implantation enables other investigators to reproduce rotenone toxicity constantly. To explore behavioral activity of rotenone-treated mice, we performed open-filed, rotarod, and cylinder test. All assessment indicated behavioral dysfunction in rotenone-treated mice. Because osmotic mini pumps were implanted under the skin on the back of each animal according to the previous report [16], the total distance was significantly decreased 1 day after implantation in all mice. To evaluate motor coordination and balance, we performed fixed-speed rotarod test (24 rpm, cutoff time 300 s). Although all mice practiced walking on the rotating rod several times prior to data collection, there was a difference in walking ability of mice. So, we examined change from value at pre-implantation of pump as shown in Figure 1D. There was no difference in value at Day 3 between control and rotenone-treated group. In control mice, time of walking on the rotating rod was extended, but not in rotenone-treated mice. We suppose that control mice got better and better through the repeated assessment, but rotenone-treated mice could not. In this study, we performed cylinder test to explore movement of both forelimbs and hindlimbs [26]. Because rotenone affects motor behavior bilaterally, the number of rearing with forelimbs contacts against the wall was measured for 2-min period irrespective of right and left paw. The number of rearing was decreased in rotenone-treated mice. Taken together, these data suggest that chronic exposure to low-dose rotenone induced motor deficits.

Next, we examined whether chronic subcutaneous injection of a low dose of rotenone (2.5 mg/kg/day) induced neurodegeneration accompanied by α-Syn accumulation in both CNS and ENS, where PD pathology was reported. In a preliminary experiment, chronic injection of rotenone (1 mg/kg/day) failed to induce dopaminergic neuronal loss in the SNpc (data not shown). We previously demonstrated that rotenone (2.5 mg/kg/day)-treated mice exhibited neurodegeneration of not only nigrostriatal dopaminergic neurons but also intestinal myenteric plexus [25]. In the present study, we could reproduce neurodegenerative effects of low-dose rotenone exposure. In addition, we observed significant reduction of cholinergic neurons in the DMV in rotenone-treated mice. Interestingly, the ChAT-positive signal in the vagal nerve was dramatically decreased in the rotenone model. Moreover, α-Syn was accumulated in neuronal soma in the SNpc, DMV and intestinal myenteric plexus, but not the hypoglossal nucleus, in our rotenone model, that is coincided with neurodegeneration. As mentioned above, Braak et al. [1] reported that PD pathology within the CNS appeared first in the olfactory bulb and the DMV, and then spread to the upper brainstem and cortical areas. In addition, several reports have demonstrated that PD pathology is also detected within the ENS [2,3,4]. Constipation is a prominent non-motor feature of PD and precedes the presentation of motor symptoms [5,6,7]. Recently, a large-scale prospective study demonstrated that lower bowel movement frequencies predicted the future PD crisis [31]. Taken together with these observations, DMV can be a key area as a junction of progression of PD pathology from ENS to CNS via vagal nerve. Correspondingly, we observed GI dysfunction in rotenone-injected mice. Therefore, our rotenone model can reproduce systemic features of PD.

Finally, we measured mitochondrial complex I activity in the nigrostriatal pathway and intestine. The 4-week chronic administration of a low dose of rotenone (2.5 mg/kg/day) did not reduce complex I activity in these tissue, which is consistent with a previous report [19], rather increased in the striatum. It suggests compensatory increase in intact cells in response to complex I inhibition by rotenone. The mechanism of neurodegeneration in our model is still unknown, that is prime concern in PD research. Considering pathological change after rotenone treatment, intracellular α-Syn accumulation could correlate to neurodegenerative process induced by the pesticide; however, Lewy body-like inclusions were not apparently observed in our model. We hope that our rotenone model will contribute to investigation of PD pathogenesis in future. Furthermore, based on a consensus that both genetic and environmental factors are thought to contribute to PD pathogenesis, the rotenone model of C57BL mice could be expanded to genetic mouse models of PD, and that could be useful animal models to investigate possible interaction between pesticide exposure and genetic defects.

## 4. Materials and Methods

### 4.1. Animals

All the experimental procedures were conducted in accordance with the NIH Guide for the Care and Use of Experimental Animals and the Policy on the Care and Use of the Laboratory Animals, Okayama University, and were approved by the Animal Care and Use Committee, Okayama University (approval reference number OKU-2017058 and OKU-2019102, approved on 1 April 2017 and 1 April 2019, respectively). Male C57BL/6J mice at 7 weeks of age were purchased from Charles River Japan Inc. (Yokohama, Japan). C57BL/6J mice were housed with a 12-h light/dark cycle at a constant temperature (23 °C) and given ad libitum access to food.

### 4.2. Rotenone Treatment

Male C57BL/6J mice (8 weeks old; approximately 25 g) were subcutaneously injected with rotenone (2.5 mg/kg/day, Sigma-Aldrich, St. Louis, MO, USA) for 4 weeks using an osmotic mini pump (Alzet, #2004; Durect Corporation, Cupertino, CA, USA). The age of animal was decided based on previous reports [22,28,32]. The mean pumping rate of the Alzet osmotic mini pump was 0.25 µL/h (= 6 µL/day). Therefore, the osmotic pump was filled with 234 µL (full volume) of rotenone (10.4 mg/mL) dissolved in the vehicle solution, consisting of equal volumes of dimethylsulphoxide and polyethylenglycol. Mice were anesthetized by isoflurane inhalation. A rotenone-filled pump was implanted under the skin on the back of each mouse. Control mouse was implanted a vehicle solution-filled pump. After 4-week period, more than 50 µL of vehicle or rotenone remained in each pump.

### 4.3. Record of Body Weight

The individual body weight of mice was recorded at 7 days before (pre), 0, and 1 day, and 1, 2, 3, and 4 weeks after implantation of vehicle- or rotenone-filled pump.

### 4.4. Behavioral Assessment

Behavioral assessment including open field, rotarod, and cylinder test was performed at 1–6 days before (pre), 1, and 3 days, and 1, 2, 3, and 4 weeks after implantation of vehicle- or rotenone-filled pump. Behavioral test was conducted from 12:00 to 16:00. Mice were habituated to the testing room at least for 1.5 h prior to behavioral assessment. Experimental areas were cleaned with 70% ethanol and wiped dry before setting the next animal. All data were analyzed using video tracking system (LimeLight, Neuroscience, Inc., Tokyo, Japan).

#### 4.4.1. Open Field Test

Locomotor activity was measured using open-field test under lower lighting condition. Each mouse was placed in the center of the open-field apparatus (50 × 50 × 35 cm). Mice could move freely, and the movement of mice was recorded for 3 min (10 s after setting until 190 s).

#### 4.4.2. Rotarod Test

Rotarod test was performed to evaluate motor coordination and balance. Prior to data collection, mice trained walking on the rotating rod (Ugo Basile 7600, Gemonio, Italy) with fixed speed (24 rpm, cutoff time 300 s) several times. The time until the animal fell off the rotating rod was recorded by observers. Because of difference in walking ability of mice on the rotating rod, change from value at pre-implantation of pump was calculated individually.

#### 4.4.3. Cylinder Test

Mice were individually placed in a transparent plastic cylinder (diameter: 13 cm, height: 16 cm), and the number of rearing with forelimbs contacts against the wall of the arena was recorded for 2 min (30 s after setting until 150 s) irrespective of right and left paw.

### 4.5. Assessment of GI Function

To evaluate GI function, fecal output was measured at 4 weeks after implantation of vehicle- or rotenone-filled pump according to a previous report [33]. Animals were removed from their home cages and placed into a new cage individually. Fecal pellets were counted every 5 min, cumulative over 20 min. Fecal pellets output from each mouse were collected and weight of pellets were measured.

### 4.6. Immunohistochemistry

For immunohistochemistry using slices of the brain and intestine, mice were perfused with ice-cold saline followed by 4% paraformaldehyde (PFA) under deep pentobarbital anesthesia (70 mg/kg, i.p.) at 4 weeks after implantation of pumps. The perfused brains and intestines were removed immediately and post-fixed for 24 h or 2 h in 4% PFA, respectively. Following cryoprotection in 15% sucrose in phosphate buffer (PB) for 48 h, the brains and intestines were snap-frozen with powdered dry ice and 20-µm-thick coronal or transverse sections were cut on a cryostat. Brain slices were collected at levels containing the mid-striatum (+ 0.6 to + 1.0 mm from the bregma), the SNpc (−2.8 to −3.0 mm from bregma) and the medulla oblongata (−13.3 to −13.6 mm from the bregma).

To evaluate dopaminergic neurodegeneration, immunostaining of TH in the SNpc and striatum and DAT in the striatum was performed. Brain slices were treated with 0.5% H_2_O_2_ for 30 min at room temperature (RT), blocked with 1% normal goat serum for 30 min, and incubated for 18 h at 4 °C with a rabbit anti-TH polyclonal antibody (1:1000; Millipore, Temecula, CA, USA) diluted in 10 mM phosphate-buffered saline (PBS) containing 0.2% Triton X-100 (0.2% PBST). After washing in 0.2% PBST (3 × 10 min), slices were reacted with biotinylated goat anti-rabbit IgG secondary antibody (1:1000; Vector Laboratories, Inc., Burlingame, CA, USA) for 2 h at RT. After washing, the sections were incubated with an avidin-biotin peroxidase complex for 1 h at RT. TH-immunopositive signals were visualized by 3,3′-diaminobenzidine tetrahydrochloride (DAB), nickel ammonium sulfate, and H_2_O_2_.

To evaluate cholinergic neurodegeneration in the DMV, immunostaining of ChAT using slices of medulla oblongata was performed. Slices were treated with 0.5% H_2_O_2_ for 30 min, 1% normal rabbit serum, and then incubated for 18 h at 4 °C with a goat anti-ChAT polyclonal antibody (1:500; Millipore) diluted in 0.2% PBST. After reaction with biotinylated rabbit anti-goat IgG secondary antibody (1:1000; Vector Laboratories, Inc.) for 2 h followed by incubation with an avidin-biotin peroxidase complex for 1 h, ChAT-immunopositive signals were visualized by DAB, nickel, and H_2_O_2_.

To evaluate neurodegeneration of myenteric plexus in the intestine, intestinal sections were incubated in 1% normal goat serum for 30 min at RT, and then reacted with rabbit anti-Tubulin β III polyclonal antibody (1:100; GeneTex, Inc., Irvine, CA, USA) for 18 h at 4 °C. After washing, slices were reacted with Alexa Fluor 488-conjugated goat anti-rabbit IgG secondary antibody (1:1000; Invitrogen, San Diego, CA, USA) for 2 h at RT. The intestinal slices were then counterstained with Hoechst 33342 nuclear stain (10 µg/mL) for 2 min, washed once and mounted with Fluoromounting medium (Dako Cytomation, Glostrup, Denmark).

To evaluate α-Syn accumulation in dopaminergic neurons in the SNpc, cholinergic neurons in the DMV or myenteric plexus in the intestine, double immunostaining of α-Syn and TH, ChAT or Tubulin β III was performed, respectively. Brain sections at a level containing the SNpc were pre-treated with 70% formic acid for 10 min at RT, and then incubated in 1% normal goat serum for 30 min at RT. Slices were incubated with a mouse anti-TH monoclonal antibody (1:1000; Millipore) and a rabbit anti-α-Syn monoclonal antibody (1:500; Cell Signaling Technology, Inc., Danvers, MA, USA) for 18 h at 4 °C. After washing, slices were reacted with Alexa Fluor 594-conjugated goat anti-mouse IgG (1:1000; Invitrogen) and Alexa Fluor 488-conjugated goat anti-rabbit IgG (1:1000; Invitrogen) secondary antibodies for 2 h at RT, respectively. Brain sections at a level containing the medulla oblongata were pre-treated with 70% formic acid for 10 min, and then incubated in 1% normal donkey serum for 30 min at RT. Slices were incubated with a goat anti-ChAT polyclonal antibody (1:500; Millipore) and a rabbit anti-α-Syn monoclonal antibody (1:500; Cell Signaling Technology, Inc.) for 18 h at 4 °C. After washing, slices were reacted with Alexa Fluor 594-conjugated donkey anti-goat IgG (1:1000; Invitrogen) and Alexa Fluor 488-conjugated donkey anti-rabbit IgG (1:1000; Invitrogen) secondary antibodies for 2 h at RT, respectively. Intestinal slices were pre-treated with 70% formic acid for 10 min, and then incubated in 1% normal goat serum for 30 min at RT. Slices were incubated with a rabbit anti-Tubulin β III polyclonal antibody (1:100; GeneTex, Inc.) and a mouse anti-α-Syn monoclonal antibody (1:1000; BioLegend, San Diego, CA, USA.) for 18 h at 4 °C. After washing, slices were reacted with Alexa Fluor 488-conjugated goat anti-rabbit IgG (1:1000; Invitrogen) and Alexa Fluor 594-conjugated goat anti-mouse IgG (1:1000; Invitrogen) secondary antibodies for 2 h at RT, respectively. All slices were counterstained with Hoechst 33342 nuclear stain (10 µg/mL).

All slides were analyzed under a fluorescence microscope (BX53; Olympus Tokyo, Japan) and cellSens imaging software (Olympus), using a mercury lamp through 360–370 nm or 470–495 nm band-pass filters to excite Hoechst 33342 or Alexa Fluor 488, respectively. Light emission from Hoechst 33342 or Alexa Fluor 488 was collected through a 420 nm long-pass filter or a 510–550 nm band-pass filter, respectively. Adobe Photoshop CS4 software was used for digital amplification of the images. Localization of α-Syn and TH, ChAT or Tubulin β III signals was confirmed by confocal laser-scanning microscopy (LSM 780; Zeiss, Oberkochen, Germany). Light emitted from Hoechst 33342, Alexa Fluor 488, or Alexa Fluor 594 was collected through a 420–470 nm band-pass filter, a 500–550 nm band-pass filter, or a 570–640 nm band-pass filter, respectively. Images were taken at a magnification of 400× and recorded using the Windows-based LSM program (Zeiss).

### 4.7. Complex I Enzyme Activity Assay

Mitochondrial complex I enzyme activity in rotenone-treated mice was measured using the Complex I Enzyme Activity Microplate Assay Kit (ab109721, Abcam, Cambridge, UK), according to manufacturer’s instructions. Tissue of ventral midbrain, striatum and intestine were dissected from mice at 4 weeks after implantation of vehicle- or rotenone-filled pump. Tissue were homogenized with PBS, centrifuged at 1000× *g* for 10 min at 4 °C, and the supernatants were collected. Total protein concentrations were determined using the Bradford-based Bio-Rad Protein Assay Dye Reagent (#5000006, Bio-Rad, Richmond, CA, USA). After adjusting sample concentration, detergent was added (1/10 *v*/*v*) to samples, and then incubated on ice for 30 min. After centrifugation at 12,000× *g* for 20 min, the supernatants were collected and used for complex I activity analysis. The enzyme activity is determined by following the oxidation of NADH to NAD+ and simultaneous reduction of a dye, which leads to increased absorbance at 450 nm. Colorimetric change was monitored for 30 min at 450 nm using a microplate reader.

### 4.8. Quantification Procedures

The number of TH-immunopositive neurons in the SNpc was counted manually under a microscope at 100× magnification. The boundary between the SNpc and ventral tegmental area was defined by a line extending dorsally from the most medial boundary of the cerebral peduncle. The relative density of TH- and DAT-positive signals in the striatum was measured quantitatively using a microscope at 40× and computer-based image analysis system (NIH ImageJ 1.52q, NIH, Bethesda, MD, USA). The number of ChAT-immunopositive neurons in the DMV was counted manually under a microscope at 200× magnification. The immunoreactivity of Tubulin β III in the myenteric plexus of the intestine was analyzed under 400× magnification and quantified using cellSens imaging software (Olympus). The integrated density of each signal was calculated as follows: Integrated density = (signal density in the myenteric plexus-background density) × area of positive signal in the plexus.

### 4.9. Statistical Analyses

Data are presented as means ± standard error of the mean (SEM). All statistical analyses were performed using KaleidaGraph v4.5 software (HULINKS Inc., Tokyo, Japan). Repeated measures analysis of variance (ANOVA) was performed to evaluate the time effect on body weight and various behavior parameters between control and rotenone-treated groups. One-way ANOVA was performed to evaluate statistical significance of time-dependent change in fecal output. Two-tailed unpaired independent *t*-test was performed to compare two groups. Multiple group comparisons were performed using Fisher’s least significant difference (LSD) test. A *p*-value < 0.05 was considered statistically significant.

## Abbreviations

PD	Parkinson’s disease
CNS	central nervous system
DMV	dorsal motor nucleus of the vagus
ENS	enteric nervous system
TH	tyrosine hydroxylase
SNpc	substantia nigra pars compacta
DAT	dopamine transporter
ChAT	choline acetyltransferase
PFA	paraformaldehyde
PB	phosphate buffer
PBS	phosphate-buffered saline
PBST	PBS containing Triton X-100
RT	room temperature
DAB	3,3′-diaminobenzidine tetrahydrochloride
GI	gastrointestinal
α-Syn	α-synuclein
ANOVA	analysis of variance
SEM	standard error of the mean
